# Improved Gel Properties of Whey Protein-Stabilized Emulsions by Ultrasound and Enzymatic Cross-Linking

**DOI:** 10.3390/gels7030135

**Published:** 2021-09-09

**Authors:** Yanli Zhao, Shiqi Xue, Xinyue Zhang, Tiehua Zhang, Xue Shen

**Affiliations:** Department of Food Science, College of Food Science and Engineering, Jilin University, Changchun 130062, China; zyl690169204@163.com (Y.Z.); xsq6786@163.com (S.X.); zxyue140@163.com (X.Z.)

**Keywords:** high-intensity ultrasound, enzymatic cross-linking, whey protein, emulsion gel, gel property

## Abstract

This study investigated the effects of high-intensity ultrasound (HUS) and transglutaminase pretreatment on the gelation behavior of whey protein soluble aggregate (WPISA) emulsions. HUS pretreatment and TGase-mediated cross-linking delayed the onset of gelation but significantly increased (*p* < 0.05) the gel firmness (G′) both after gel formation at 25 °C and during storage at 4 °C. The frequency sweep test indicated that all gels had a similar frequency dependence at 4 and 25 °C, and the elasticity and viscosity of the WPISA-stabilized emulsion gel were significantly enhanced by HUS pretreatment and TGase-mediated cross-linking (*p* < 0.05). HUS and TGase-mediated cross-linking greatly improved the textural properties of WPISA-stabilized emulsion gels, as revealed by their increases in gel hardness, cohesiveness, resilience, and chewiness. HUS pretreatment and TGase-mediated cross-linking significantly increased the water-holding capacity but decreased the swelling ratios of the gels (*p* < 0.05). Interactive force analysis confirmed that noncovalent interactions, disulfide bonds, and TGase-induced covalent cross-links were all involved in the formation of gel networks. In conclusion, the combination of HUS and TGase-mediated cross-linking were beneficial for improving the gelation properties of WPISA-stabilized emulsion as a controlled release vehicle for potential food industrial applications.

## 1. Introduction

Emulsion gel, also called emulsion-filled gel or emulgel, with emulsified oil droplets entrapped within the gel matrix, has gained great attention due to its designable textural attributes and wide applications in food and nonfood fields [1]. Emulsion gels are structured emulsion systems that can simultaneously deliver hydrophobic and hydrophilic bioactive compounds and participate in food structure formation and stabilization [2]. Generally, emulsion gel can be produced by a two-step process that involves emulsification (formation of emulsion) followed by a gelation process to form three-dimensional networks of gel matrix (e.g., protein and polysaccharide). For gelation, cold-set methods are more beneficial to encapsulate, protect, and deliver heat-sensitive nutraceutical components (β-carotene, probiotic, polyphenol, and so on) from degradation during heat treatment [3]. In particular, the growing demand for protein-dense foods from animal and plant sources has contributed to more practical applications of protein-stabilized emulsion gels in food formulations.

Whey protein, a coproduct of cheese making, is extensively used in many food formulations due to its high nutritional value and desirable functional properties, including emulsifying and gelation properties [4,5]. Therefore, whey proteins are often added to foods as emulsifiers and gelling agents at the same time, and this is an ideal condition for emulsion gel formation. The formation of whey protein soluble aggregates (WPISA) gives them the ability to produce cold set gels [6]. The recognized roles of protein-stabilized oil droplets as active fillers in a protein-based gel matrix have resulted in the crucial impact of the gelling matrix on emulsion gel properties [7,8]. For example, Qayum et al. (2021) combined ultrasound pretreatment and laccase cross-linking to enhance the gel properties of α-lactalbumin emulsion gels [9]. Guo et al. (2017) made it possible to control the release of nutrients or bioactive compounds in WPI-stabilized oil-in-water emulsion gels by manipulating the gel structure [10]. For high protein/oil ratio emulsions, the gel properties largely depend on the proteins because of their major role in the continuous phase or at the oil/water interface [11]. Therefore, it is possible to tailor the gelation properties of whey protein emulsion gels by protein modification for a special purpose.

It is well known that high-intensity ultrasound (HUS, 10–1000 W cm^−2^, with a frequency of 20–100 kHz) can alter food protein properties through acoustic cavitation. A high amount of highly localized energy is generated during the collapse of cavitation bubbles in liquids [12,13]. We recently used high-intensity ultrasound (HUS, 20 kHz) to facilitate the transglutaminase-mediated (EC 2.3.2.13, TGase) cross-linking reaction of whey protein soluble aggregates. HUS pretreatment successfully increased the TGase-mediated cross-linking degree and improved the gel properties of TGase cross-linked WPISA hydrogels [14]. Compared to hydrogels, emulsion gels can work as both emulsions and hydrogels because of their structure containing a gelling matrix and dispersed oil droplets. Furthermore, emulsion gels have a number of potentially practical applications in foods, cosmetics, and pharmaceuticals; in turn, the structural and release properties are more designable and controllable. To the best of our knowledge, there is limited information about the application of HUS to modify the gelation properties of TGase-catalyzed WPISA and its underlying mechanism, especially for emulsion gel delivery systems. Therefore, the objective of this study was to investigate the effect of HUS (20 kHz, ~69 Wcm^−2^, 5–15 min) on the gelation properties of glucono-δ-lactone (GDL)-induced transglutaminase cross-linked WPISA emulsion gels.

## 2. Results and Discussion

### 2.1. Emulsion Characterization

**Figure 1:**
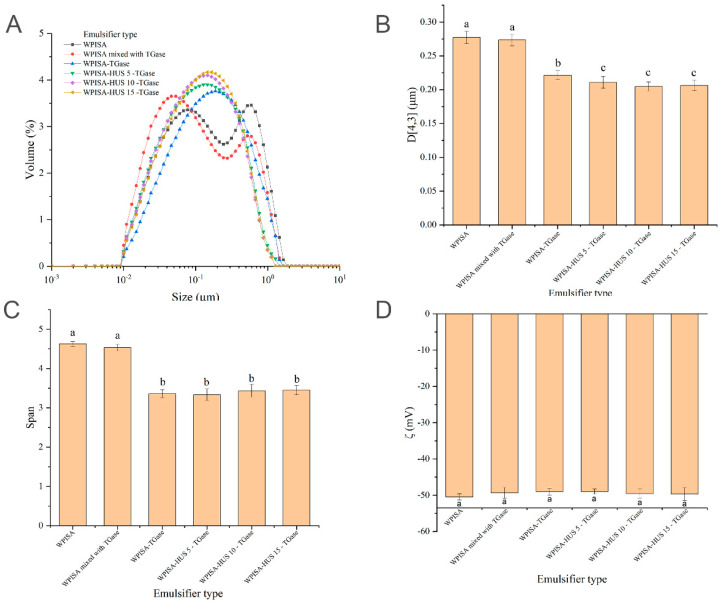
Effects of HUS pretreatment and TGase cross-linking on size distribution (**A**), volume mean size D[4,3] (**B**), span (**C**), and zeta potential (**D**) of WPISA-stabilized emulsions at pH 7.0. WPISA: untreated whey protein soluble aggregates, WPISA mixed with TGase: a mixture of WPISA and inactivated TGase without cross-linking reaction, WPISA-TGase: WPISA cross-linked by TGase, WPISA-HUS 5-TGase: WPISA pretreated by ultrasound for 5 min before TGase cross-linking, WPISA- HUS 10-TGase: WPISA pretreated by ultrasound for 10 min before TGase cross-linking, WPISA- HUS 15-TGase: WPISA pretreated by ultrasound for 15 min before TGase cross-linking. Values with different lowercase letters indicate significant differences (*p* < 0.05).

The droplet size distributions of the emulsions stabilized by six emulsifiers (WPISA, WPISA mixed with TGase, WPISA-TGase, WPISA-HUS 5-TGase, WPISA- HUS 10-TGase, and WPISA- HUS 15-TGase) are shown in Figure 1A. Emulsions stabilized by WPISA and WPISA mixed with TGase had bimodal distributions and submicron droplet sizes (Figure 1B), with span values greater than 4.5 (Figure 1C). Emulsions stabilized by WPISA-HUS (0, 5, 10, 15)-TGase had monomodal size distributions with significantly smaller span values than emulsions stabilized by WPISA (Figure 1C). TGase-mediated cross-linking resulted in a significant reduction in the D[4,3] of oil droplets from 0.277 ± 0.009 to 0.221 ± 0.007 μm (*p* < 0.05). The decrease in D[4,3] of oil droplets might be because the formation of TGase cross-linked WPISA polymers induced a decrease in protein solubility [14] and limited the immediate absorbance of protein onto the oil-water interface to form small droplets in the emulsion. Similarly, the combination of microfluidization and TGase cross-linking was found to cause a droplet size reduction in peanut protein isolate-stabilized emulsions [15]. HUS pretreatment significantly decreased the D[4,3] of emulsions stabilized by TGase cross-linked WPISA (*p* < 0.05), which may be due to the promotion effect of HUS on cross-linking reactions resulting in the formation of more TGase cross-linked polymers [14]. The zeta potentials of the emulsions stabilized by six emulsifiers are shown in Figure 1D. All of the emulsions were negatively charged, which may be attributed to the pH being above the pI of whey proteins. The absolute values of the zeta potential were all higher than 49 mV, which is considered relatively stable [16]. The combination of HUS and TGase-mediated cross-linking did not significantly change the zeta potential of the WPISA-stabilized emulsion (*p* > 0.05).

### 2.2. Rheological Properties

#### 2.2.1. Gelation Kinetics

**Figure 2:**
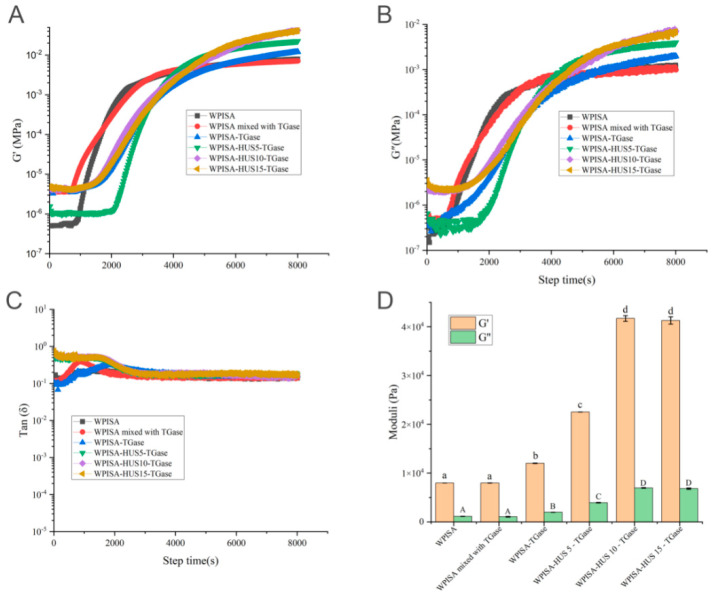
Evolution of the (**A**) storage modulus (G′), (**B**) loss modulus (G″), (**C**) loss factor (tan *δ*), and (**D**) moduli at 8000 s of emulsion gels stabilized by WPISA, WPISA mixed with TGase, WPISA-TGase, WPISA-HUS5-TGase, WPISA-HUS10-TGase, and WPISA-HUS15-TGase. Values with different lowercase letters indicate significant differences (*p* < 0.05).

Small amplitude oscillatory rheology was used to characterize the rheological properties during gel formation. The gelation behavior of TGase cross-linked WPISA emulsion gel upon HUS pretreatment is shown in Figure 2. When the temperature was maintained at 25 °C, the emulsion gel was formed as a function of time after the addition of GDL. The gel formation kinetics of all emulsion gels showed three obvious stages during the tested times (8000 s). The values of G′ and G″ had similar trends during gel formation. At the initial stage, both G′ (Figure 2A) and G″ (Figure 2B) increased slowly, indicating that the interactions between WPISA occurred gradually with the hydrolysis of GDL increasing in the systems, resulting in the pH being reduced slowly and still being higher than the pI of whey proteins (4.6–5.1) [8]. At the second stage, both G′ and G″ showed sharp increases (G′: 10^−5^ - > 10^−3^ MPa; G″: 10^−5^ - > 10^−3^ MPa), and G′ remained higher than G″, which indicated that the 3D networks were formed due to the formation of disulfide bonds and noncovalent interactions (e.g., hydrophobic interactions, ionic bonds, van der Waals forces, and hydrogen bonding) between protein particles and rearrangements in the protein networks [17].

Generally, gelation time refers to the time point at which the G′ and G″ moduli crossover. However, all of the emulsion systems (mixed with GDL) showed that G′ was already higher than G″ at the beginning of the rheological test (tan *δ* < 1, Figure 2C), probably because of rapid decreases in the pH (from 6.9 to 6.0–6.2) of the emulsion system after GDL addition, which was consistent with a previous study [18]. The reduction in pH may be enough to induce a weak gel network of gel matrix (WPISA) before data acquisition started by the rheometer [19,20]. Therefore, the time point at which the dramatic increase started in the G′ value was considered the gelation time (onset gelation) [20]. There was no apparent change in the gelation time between emulsion gels stabilized by WPISA and WPISA mixed with TGase. However, the TGase cross-linked WPISA (with or without HUS pretreatment)-stabilized emulsion showed a longer gelation time than the WPISA-stabilized emulsion, suggesting that the presence of TGase cross-linked WPISA in the emulsion system delayed the onset gelation of the WPISA-stabilized emulsion. In the last stage, G′ and G″ increased slowly with increasing holding time until 8000 s, as explained by the almost steady state of the gel network developed under a low pH value.

At the end of the test time (8000 s), the emulsion gel stabilized by TGase cross-linked WPISA had significantly higher G′ and G″ values (*p* < 0.05) than those of the emulsion gel stabilized by WPISA (Figure 2D). Earlier studies have assumed that TGase-mediated covalent cross-linked whey protein molecules could both absorb to the surfaces of oil droplets and distribute in the surrounding continuous phase, thereby affecting the rheology and stability of the emulsion gels [21]. HUS treatment (5, 10, and 15 min) significantly increased (*p* < 0.05) the G′ and G″ values of TGase cross-linked WPISA-stabilized emulsion gels compared with those without HUS-treated counterparts, which was mainly attributed to the positive facilitating impact of HUS on the TGase cross-linking reaction. HUS treatment can cause partial unfolding of WPISA to expose some active/functional groups to TGase, thereby leading to the formation of more TGase cross-lined WPISA, which was responsible for the increases in the G′ and G″ values of emulsion gels [18]. G′, an indicator of gel firmness, can reflect the elastic/solid-like properties of an emulsion gel [8]. Therefore, HUS treatment and TGase-mediated cross-linking increased the G′ values of the emulsion gel, probably because of the formation of TGase cross-linked WPISA, which may contribute to forming a compact gel network and increasing the gel firmness. These results suggest that HUS is an efficient method to increase the amount of TGase cross-linked WPISA and therefore increase the emulsion gel firmness.

#### 2.2.2. Viscoelastic Properties during Frozen Storage

**Figure 3:**
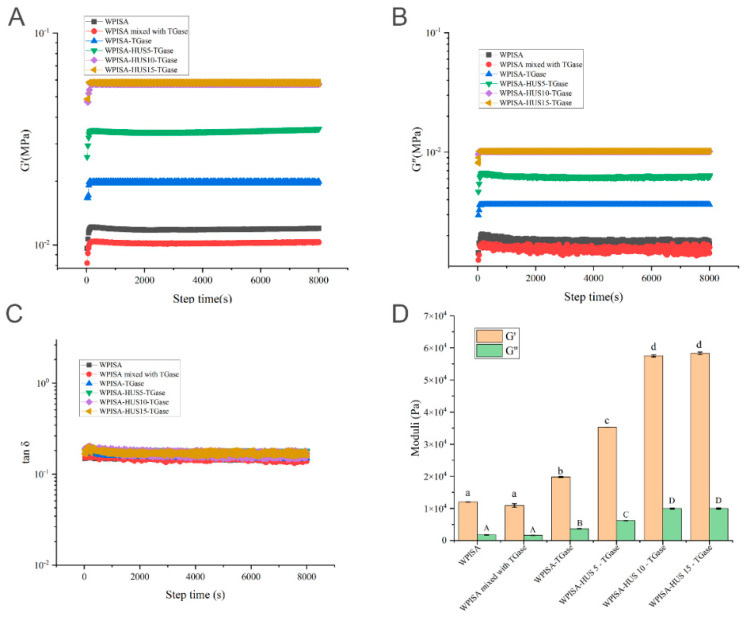
Effects of HUS pretreatment and TGase cross-linking on the gelation profile of the GDL induced WPISA emulsion gels during storage at 4 °C for 8000 s. (**A**): storage modulus (G′), (**B**): loss modulus (G″), (**C**):loss factor (tan *δ*), and (**D**): moduli at 8000 s. Values with different lowercase letters indicate significant differences (*p* < 0.05).

Another sequential 8000 s sweep test was performed after the first 8000 s at 25 °C to evaluate the rheological properties of emulsion gels during storage at 4 °C. Both the G′ and G″ of all samples showed sharp increases at first and remained unchanged (Figure 3A,B), suggesting a plateau at which the gel had a very stable state during refrigerated storage. Loss factor (tan *δ*) values were in the range of 0.15–0.25 (Figure 3C), suggesting that G′ was approximately 5-fold higher than G″. After TGase cross-linking, the WPISA-stabilized emulsion gel had significantly higher G′ and G″ (*p* < 0.05) (Figure 3D), which indicated the presence of more covalent bonds contributing to the increase in the G′ values. These results were consistent with the findings reported by Sivan et al. [22], who found that the enzymatically cross-linked soy protein contains covalent bonds, resulting in a higher G′ value at the end of the gelation process. Furthermore, HUS treatment (5, 10, and 15 min) significantly increased (*p* < 0.05) the G′ and G″ values of TGase cross-linked WPISA-stabilized emulsion gels as a result of the improvement of HUS on the TGase cross-linking degree.

#### 2.2.3. Frequency Dependence of Emulsion Gel

**Figure 4:**
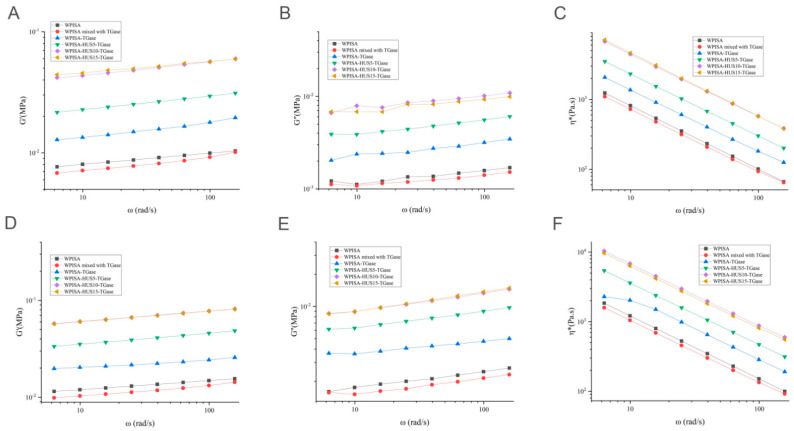
Frequency dependence of (**A**,**D**) storage modulus (G′), (**B**,**E**) loss modulus (G″), and (**C**,**F**) complex viscosity (η*) of TGase cross-linked WPISA emulsion gels under different sonication time.

Over the entire frequency range, all gel samples showed a typical viscoelastic solid (elastic, solid-like) behavior with a gel-like structure, as demonstrated by G′ > G″ (Figure 4A,B,D,E) and a linear reduction in η* (Figure 4C,F). The G′ and G″ values of all gel samples showed a positive relation with increasing frequency from 5 to 150 rad/s. The frequency sweep test confirmed the firmer texture of the TGase cross-linked stabilized emulsion gel with greater G′ values than the WPISA-stabilized emulsion gel. HUS treatment (10 and 15 min) made the emulsion gel firmer due to its facilitating effect on the TGase cross-linking reaction. As expected, these results suggest that the HUS-induced increase in the degree of TGase-catalyzed cross-linking in WPISA was the main reason for the improved emulsion gel firmness.

### 2.3. Textural Properties

Gel food undergoes compression and stretching to break down its structure inside the mouth during oral processing, which is vital to our eating experience and sensory perception [23]. Texture appreciation accompanies the entire mastication process. TPA tests can simulate the chewing process [24]. Table 1 shows the textural characteristics of the TGase cross-linked WPISA-stabilized emulsion gels upon HUS pretreatment. Hardness can reflect the strength of a gel structure under compression [25]. There was a significant increase (*p* < 0.05) in the hardness of the WPISA-stabilized emulsion gel after cross-linking by TGase. However, the addition of TGase to WPISA before emulsion formation had no significant impact on the hardness of the WPISA-stabilized emulsion gel (*p* > 0.05). This result indicated that the TGase-mediated cross-linking reaction among whey protein molecules was responsible for increasing the hardness of the emulsion gel. HUS pretreatment (10 and 15 min) significantly increased the hardness of TGase cross-linked WPISA-stabilized emulsion gel, suggesting that HUS facilitated the cross-linking reaction resulting in the formation of a dense gel network with a firmer texture to whey protein emulsion gel. Therefore, the increased hardness of the emulsion gel possibly resulted from more TGase cross-linked WPISA in the gel matrix, as HUS was beneficial for improving the TGase cross-linking reaction.

Similar to hardness, HUS pretreatment (10 and 15 min) had a significant impact (*p* < 0.05) on the resilience, chewiness, and cohesiveness of TGase-catalyzed WPISA-stabilized emulsion gel. These results may be related to the TGase-mediated cross-linking reaction that favors dense network formation and leads to a better gel structure.

### 2.4. WHC and Swelling Ratio

**Figure 5:**
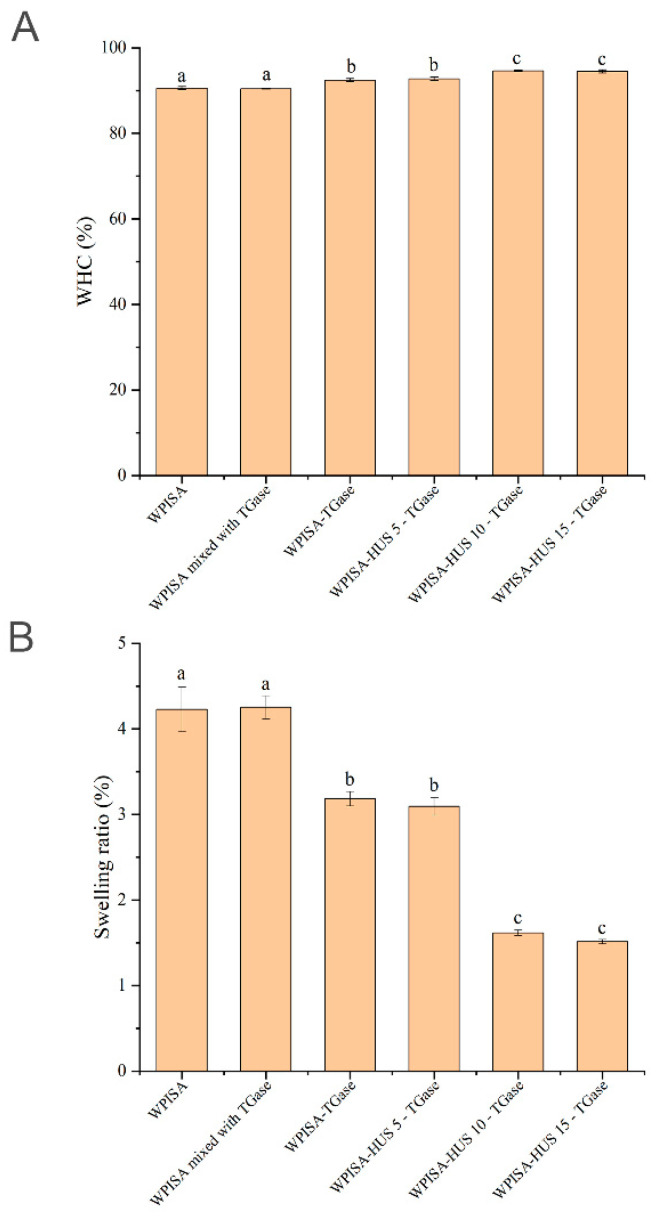
Effects of ultrasound pretreatment on water-holding capacity (**A**) and swelling ratio (**B**) of TGase cross-linked WPISA-stabilized emulsion gel. Values with different lowercase letters indicate significant differences (*p* < 0.05).

Water-holding capacity refers to the ability of gels to stabilize water molecules through capillary effects, which are closely related to pore size [19]. As shown in Figure 5A, all of the samples had high WHC values (>90%), and there were small differences among the samples. The WHC of the GDL-induced WPISA-stabilized emulsion gel increased significantly from 90% to 92% after incubation with TGase for 4 h (Figure 5A). There was no significant change (*p* > 0.05) in the WHC of the WPISA-stabilized emulsion gel after mixing with WPISA and TGase before emulsion formation. These results indicated that TGase-mediated cross-linking reactions among WPISA molecules might contribute to the increase in the WHC of the emulsion gel. TGase-mediated cross-linking has been reported to improve the WHC of hydrogels produced from several food proteins, including soy proteins [18], faba bean protein isolate [26], pea proteins [27], and whey proteins [28]. HUS pretreatment (10 and 15 min) significantly increased (*p* < 0.05) the WHC of the WPISA-stabilized emulsion gel after incubation with TGase, which indicated that HUS pretreatment had a beneficial effect on the TGase-mediated cross-linking reaction to facilitate the formation of a dense gel network in favor of holding more water molecules within the gel matrix strongly against centrifugation. It has been reported that emulsion gels with higher WHC values may benefit from denser and more uniform microstructures, as demonstrated by a higher storage modulus [29]. This finding agrees with some previous studies [29,30], which reported that WHC was positively correlated with the storage modulus of emulsion gels resulting from their ability to hold water molecules against centrifugation.

The 3D network in emulsion gels contributes to both water entrapment and adsorption of additional water from the surrounding environment [31]. It can impact the mouthfeel of gel food and the release of incorporated bioactives [7]. The swelling property of gel was closely related to the pore size of the network and the water-adsorbing capacity of the gelling biopolymers [32]. Figure 5B shows the swelling behavior of TGase cross-linked WPISA emulsion gel upon HUS treatment. The swelling ratio of the WPISA emulsion gel significantly decreased after TGase cross-linking (*p* < 0.05). The smaller adsorption of water might result from a more compact gel network and more adsorbed water in the gel during the development of the gel network. Moreover, the TGase-mediated cross-linking reaction was beneficial for exposing the hydrophobic amino acid residues of WPISA, reducing water uptake, and therefore decreasing swelling ratios [14]. HUS treatment (10 and 15 min) significantly decreased the swelling ratio of the TGase cross-linked WPISA emulsion gel, which may be due to the increased degree of TGase cross-linking in WPISA, the modified interactions among the gel matrix, and changes in the 3D structure of the emulsion gel. These results indicated that the increase in cross-linking degree might make the network structure more compact, which was not favorable for water penetration. This was probably responsible for the decrease in the swelling ratio of the WPISA emulsion gels.

### 2.5. Gel Solubility in Various Solvents

The protein solubility of the emulsion gel was applied as an indication of its ability to be dispersed in different solvents. Higher protein solubility indicates higher dispersity of the gels in the solvent and weaker or fewer bonds involved within the gel networks [18]. The differences in protein solubility, represented as B-A, C-B, D-C, and E-D, can reflect how much the interactive forces (electrostatic forces, hydrophobic interaction, hydrogen bonding, and disulfide bonding) contribute to the gel networks. All of the gel samples had very small protein solubility values in solvent A, which were negligible compared to other solvents. As shown in Table 2, the protein solubility of emulsion gels increased with the solvent order, A < B < C < D < E, indicating that the increasing magnitude interactive forces were involved in emulsion gel network formation and/or maintenance because of the disruption effect of buffers against their target bond [33]. Especially in solvent E, the protein solubility of the WPISA-stabilized emulsion gel decreased significantly (*p* < 0.05) after incubation with TGase. However, WPISA mixed with TGase before emulsion formation had no significant impact on the protein solubility of the WPISA-stabilized emulsion gel (*p* > 0.05). These results indicated that the TGase-mediated cross-linking reaction among WPISA molecules was responsible for the decrease in the protein solubility of the emulsion gel, and the TGase-induced covalent cross-links contributed to the formation of gel networks. HUS pretreatment (10 and 15 min) significantly decreased the protein solubility of TGase cross-linked WPISA-stabilized emulsion gel, which indicated that HUS facilitated the formation of more TGase covalent cross-linked WPISA resulting in insensitivity to solvent E. TGase cross-linked whey proteins were formed through intramolecular and/or intermolecular cross-linking reactions by isopeptide bond formation. The ε-(γ-glutamyl) lysine isopeptide bond was reported to be insensitive to 2-mercaptoethanol [34].

The distributions of intermolecular forces within gel samples were in the following order: hydrophobic interactions > disulfide bonds > hydrogen bonds ≥ electrostatic interactions (Table 2), indicating that hydrophobic interactions and disulfide bonds played a major role in the acid-induced WPISA emulsion gels regardless of TGase treatment. The changes in electrostatic interactions and hydrogen bonds of WPISA-stabilized emulsion gels showed a similar trend after incubation with TGase upon HUS pretreatment. The electrostatic interactions and hydrogen bonds of WPISA emulsion gels decreased significantly (*p* < 0.05) after incubation with TGase. HUS pretreatment (10 and 15 min) significantly decreased the electrostatic interactions and hydrogen bonds in TGase cross-linked WPISA-stabilized emulsion gel, indicating that the increase in cross-linking degree was responsible for the reduction of electrostatic interactions and hydrogen bonds. The hydrophobic interactions in emulsion gels were increased significantly by HUS pretreatment (10 and 15 min) (*p* < 0.05). There was no change in protein solubility (E-D) among all gel samples, indicating that HUS and TGase-mediated cross-linking reactions had no significant impact on disulfide bond formation in gel networks.

## 3. Conclusions

It was feasible to combine high-intensity ultrasound and TGase-mediated cross-linking to improve the acid-induced gelation properties of whey protein emulsion gels. The combination of HUS and TGase cross-linking increased the gel firmness (G′), hardness, cohesiveness, resilience, chewiness, and WHC and decreased the swelling ratios of WPISA-stabilized emulsion gels. These results indicated that the improvement effect of HUS on TGase-catalyzed cross-linking played an essential role in the formation of 3D gel networks by introducing new covalent bonds in WPISA. A combination of HUS and TGase cross-linking may facilitate the design of whey protein-based emulsion gels for applications related to the controlled release of nutrients or bioactive compounds. The protection and controlled-release properties of bioactive ingredients need to be determined in future works.

## 4. Materials and Methods

### 4.1. Materials

Whey protein isolate (WPI, 93.14%) was purchased from Fonterra (Auckland, New Zealand). Transglutaminase (200 U g^−1^ enzyme activity) was purchased from Yuanye Biological Technology Co., LTD (Shanghai, China). Corn oil was purchased from a local supermarket. Glucono-δ-lactone (GDL) was purchased from Hui Yang Biological Technology Co., (Dezhou, China). All other chemicals used in this study were reagent grade and purchased from Sigma (St. Louis, MO, USA). The water used in this study was filtered using a Millipore Milli-Q™ water purification system (Millipore Corp., Milford, MA, USA).

### 4.2. Preparation of TGase-Catalyzed Whey Protein Soluble Aggregates and Ultrasound Protocol

TGase-catalyzed WPISA solution (10%, *w*/*v*) was prepared as we previously described [14]. Briefly, WPISA was prepared by preheating WPI solution (10%, *w*/*v*; protein: 9.31% *w*/*v*) at 80 °C for 15 min (pH 7.0) while stirring (1500 rpm) in a water bath. Ultrasound treatment (20 kHz, 0, 5, 10, and 15 min, 30% amplitude, ~69 W cm^−2^) was performed in 10 s: 5 s work/rest cycles using an ultrasonic processor (VCX800, vibra cell, Sonics, USA). Samples were immersed in an ice-water bath to remove the heat generated by ultrasound treatment. HUS treated samples were incubated with TGase (10 U g^−1^ protein) at 50 °C for 4 h while stirring. TGase was inactivated by heating, and the samples were cooled to room temperature for further use.

Samples treated by ultrasound and TGase were marked as follows:

WPISA (untreated), WPISA mixed with TGase (a mixture of WPISA and inactivated TGase without cross-linking reaction), WPISA-TGase (WPISA cross-linked by TGase), WPISA-HUS 5-TGase (WPISA pretreated by ultrasound for 5 min before TGase cross-linking), WPISA-HUS 10-TGase (WPISA pretreated by ultrasound for 10 min before TGase cross-linking), and WPISA-HUS 15-TGase (WPISA pretreated by ultrasound for 15 min before TGase cross-linking).

### 4.3. Preparation of Emulsion Gels

Oil-in-water emulsions were prepared by mixing the protein solution (water phase, WPISA, WPISA mixed with TGase, WPISA-TGase, WPISA-HUS 5-TGase, WPISA- HUS 10-TGase, and WPISA- HUS 15-TGase) and corn oil at a ratio of 9:1 (*v*/*v*). Sodium azide (0.022%, *w*/*v*) was added to the aqueous phase to inhibit the growth of the microorganism. The emulsion was formed using an Ultra-Turrax T25 high-speed blender (IKA, Staufen, Germany) at 12,000 rpm/min for 2 min, followed by homogenization using an ultrasonic processor (VCX800, Vibra cell, Sonics, Newtown, CA, USA) at 40% amplitude for 5 min (10 s/5 s work/rest cycles). GDL (1%, *w*/*v*) was added into the emulsions with gentle stirring for 2 min. The mixtures were maintained at 25 °C for 2 h for gel formation. Then, the gels were stored at 4 °C for 8 h until further analyses.

### 4.4. Characterization of Emulsions

#### 4.4.1. Particle Size Distribution

Particle size was determined by laser diffraction using a Mastersizer 3000 (Malvern Instruments Ltd., Malvern, Worcestershire, UK) equipped with a wet sample dispersion unit (Malvern Hydro MV, UK). The refractive index of corn oil and water was 1.47 and 1.330, respectively. The samples were added into a circulating cell until an obscuration value within 8–12%. Four diameters (D_v_90, D_v_10, D_v_50, and D[4,3]) were used to analyze the system [35]. The volume-weighted mean diameter D[4,3] was used as a representative mean droplet size to interpret the results. The results reported as the average and standard deviation of measurements made on at least three freshly prepared samples, with three readings made per sample. Span was used to express the width of the sample size distribution, which was calculated as follows:

Span = (D_v_90 − D_v_10)/D_v_50 (1)

where, D_v_90, D_v_10, and D_v_50 are diameters below which are 10%, 50%, and 90% of the overall droplet volume, respectively.

#### 4.4.2. Zeta-Potential

Zeta-potential (ζ, mV) of the emulsions were measured using a Zetasizer Nano ZS 90 (Malvern Instruments, Malvern, UK). The emulsions were diluted (0.5%, *v*/*v*) with 0.01 M phosphate buffer (pH7.0) and analyzed at a scattering angle of 173°. ζ was calculated based on the Henry equation [36].

### 4.5. Determination of Rheological Properties

The gelation process of emulsion gels was observed using a DHR-1 rheometer (TA Instruments, New Castle, DE, USA) equipped with a steel parallel plate geometry (40 mm diameter, 1 mm gap). The emulsions were mixed with GDL (1%, *w*/*v*) before rheological tests. A thin layer of glycerin was applied to prevent water evaporation. The temperature was controlled by a Peltier unit attaching a water circulation system.

#### 4.5.1. Gel Formation and Refrigeration Process

A time sweep test was performed for 8000 s to study the gelation process at an angular frequency of 10 rad/s at 25 °C. The strain was set to 1% within the linear viscoelastic region (LVR) basing on the oscillatory amplitude analyses. The storage (G′) and loss (G″) moduli and loss factor (tan *δ*) were recorded during the test. Another 8000 s sweep test was performed subsequently at 4 °C to simulate the refrigeration process.

#### 4.5.2. Frequency Sweep Test

A frequency sweep test was conducted at a strain of 1% over the 1–100 Hz range at 25 °C. The storage modulus (G′), loss modulus (G″), and complex viscosity (η*) were all recorded.

### 4.6. Determination of Gelation Properties

#### 4.6.1. Textural Properties

For textural analysis, emulsion gels were formed in 3.4 mL plastic cups (16 mm internal diameter×17 mm height), as described above. Texture profile analysis (TPA) test was performed on a Brookfield CT3 texture analyzer (Brookfield, Middleboro, MA, USA) with a cylindrical plunger (diameter = 12.7 mm) and a 1000 g compression head. Gel samples were compressed at a constant speed of 0.5 mm/s with a 2 mm test distance. The parameters determined were hardness, resilience, chewiness, cohesiveness, and springiness.

#### 4.6.2. Water-Holding Capacity

The water-holding capacity (WHC) of emulsion gels was determined according to a method [37] with some modifications. Emulsion gels were formed in 10 mL centrifuge tubes, centrifuged (Avanti J-E, Beckman, Brea, CA, USA) at 8000× *g* for 30 min at 4 °C. The released water was drained and weighed. WHC was calculated as the ratio of the weight of gel remaining in the centrifuge tube to the initial emulsion gel weight.

#### 4.6.3. Swelling Ratio

Swelling ratio of the gels was determined according to a literature method [2], with some modifications. Briefly, a cylindrical emulsion gel sample was formed in a disposable syringe without a needle with a volume of 5 mL. The gel samples were cut into pieces and weighed (recorded as W_0_). The gel samples were immersed in deionized Milli-Q water for 24 h at room temperature. The samples were taken out, blotted to remove surface water with filter paper carefully, and weighted (recorded as W). The swelling ratio was calculated using the following equation:

Swelling ratio = (W − W_0_)/W_0_ × 100% (2)

#### 4.6.4. Solubility Measurements

The protein solubility of the emulsion gels in different solvents was measured according to a method [38] with some modifications. The emulsion gels were dispersed in distilled water (buffer A), Tris-glycine buffer (pH 8.0) (buffer B), B supplemented with 1% (*w*/*v*) sodium dodecyl sulfate (buffer C), C supplemented with 8 M urea (buffer D), and D supplemented with 1% (*v*/*v*) 2-mercaptoethanol (2-ME) (buffer E), respectively. The emulsion gel samples were incubated with different solvents at 37 °C in a shaking water bath for 12 h, centrifuged at 10,000× *g* for 15 min (25 °C). The protein concentration was determined using a bicinchoninic acid (BCA) protein assay kit (Beyotime, Shanghai, China). The protein solubility (%) was calculated as follows: The measured protein content divided by the total protein content and multiplied by 100%. The interaction forces in gels were expressed as solubility difference between B and A (electrostatic interaction), C and B (hydrophobic interaction), D and C (hydrogen bonds), and E and D (disulfide bonds), respectively [39].

### 4.7. Statistical Analyses

All experiments were performed in triplicates. Statistical analyses were performed using IBM SPSS Statistics 23. A One-way ANOVA and post-hoc analyses were used to compare the data of different groups, where LSD method and Dunnet’C were used on the basis of the homogeneity test. The data were plotted by Origin software (OriginPro 2021, Originlab Corporation, Northampton, MA, USA). The results were expressed as mean ± standard deviation (SD) and considered significantly different when *p* < 0.05 at 95% level of confidence.

## Figures and Tables

**Table 1:** Texture profile analyses of WPISA stabilized emulsion gel pretreated with HUS and TGase cross-linking.

	Hardness	Cohesiveness	Resilience	Chewiness
	(g)			(mJ)
WPISA	611.8 ± 6.458 ^a^	0.67 ± 0.015 ^a^	0.22 ± 0.012 ^a^	7.90 ± 0.085 ^a^
WPISA mixed with TGase	624.5 ± 9.151 ^a^	0.66 ± 0.044 ^a^	0.22 ± 0.012 ^a^	8.01 ± 0.110 ^a^
WPISA-TGase	670.8 ± 17.21 ^b^	0.69 ± 0.006 ^a^	0.24 ± 0.001 ^b^	8.53 ± 0.165 ^b^
WPISA-HUS 5-TGase	687.8 ± 23.72 ^b^	0.71 ± 0.017 ^a^	0.25 ± 0.015 ^b^	8.92 ± 0.377 ^c^
WPISA-HUS 10-TGase	774.8 ± 7.433 ^c^	0.79 ± 0.04 ^b^	0.27± 0.001 ^c^	9.43 ± 0.227 ^d^
WPISA-HUS 15-TGase	795.6 ± 7.862 ^c^	0.78 ± 0.03 ^b^	0.26667 ± 0.001 ^c^	9.71 ± 0.133 ^d^

WPISA: untreated whey protein soluble aggregates, WPISA mixed with TGase: a mixture of WPISA and inactivated TGase without cross-linking reaction, WPISA-TGase: WPISA cross-linked by TGase, WPISA-HUS 5-TGase: WPISA pretreated by ultrasound for 5 min before TGase cross-linking, WPISA- HUS 10-TGase: WPISA pretreated by ultrasound for 10 min before TGase cross-linking, WPISA-HUS 15-TGase: WPISA pretreated by ultrasound for 15 min before TGase cross-linking. Different superscript letters in the same column indicate significant difference among the values at the 95% confidence level (*p* < 0.05).

**Table 2:** Difference in the relative solubility of protein pretreated with HUS and TGase cross-linking in the emulsion gels between various solvents.

	A (%)	B-A (%)	C-B (%)	D-C (%)	E-D (%)	E (%)
WPISA	0.113 ± 0.011 ^a^	9.97 ± 0.46 ^a^	23.87 ± 1.02 ^a^	13.92 ± 1.38 ^a^	19.24 ± 1.70 ^a^	67.10 ± 2.34 ^a^
WPISA mixed with TGase	0.106 ± 0.006 ^a^	9.97 ± 0.64 ^a^	23.79 ± 0.49 ^a^	13.91 ± 0.13 ^a^	19.13 ± 0.88 ^a^	66.86 ± 0.76 ^a^
WPISA-TGase	0.114 ± 0.009 ^a^	9.04 ± 0.68 ^b^	23.90 ± 0.92 ^a^	9.60 ± 0.87 ^b^	19.03 ± 0.98 ^a^	62.82 ± 1.03 ^b^
WPISA-HUS 5-TGase	0.113 ± 0.007 ^a^	8.89 ± 0.31 ^b^	24.07 ± 0.76 ^a^	9.17 ± 0.71 ^b^	18.67 ± 1.44 ^a^	61.97 ± 2.03 ^b^
WPISA-HUS 10-TGase	0.108 ± 0.010 ^a^	7.67 ± 0.21 ^c^	25.47 ± 0.69 ^b^	7.37 ± 0.99 ^c^	19.02 ± 1.02 ^a^	57.65 ± 1.46 ^c^
WPISA-HUS 15-TGase	0.119 ± 0.015 ^a^	7.32 ± 0.27 ^c^	25.60 ± 0.47 ^b^	7.53 ± 0.67 ^c^	18.96 ± 0.45 ^a^	57.36 ± 1.10 ^c^

Solvents: distilled water (buffer A), Tris-glycine buffer (pH 8.0) (buffer B), B supplemented with 1% (*w*/*v*) sodium dodecyl sulfate(C), C supplemented with 8 M urea (D), D supplemented with 1% (*v*/*v*) 2-mercaptoethanol (2-ME) (E). Interaction forces: electrostatic interaction (B-A), hydrophobic interaction (C-B), hydrogen bonds (D-C), disulfide bonds (E-D). Different superscript letters in the same column indicate significant difference among the values at the 95% confidence level (*p* < 0.05).

## Data Availability

The data presented in this study are available on request from the corresponding author.
